# Supplementary material for: One-Pot Synthesis of Double-Network PEG/Collagen Hydrogel for Enhanced Adipogenic Differentiation and Retrieval of Adipose-Derived Stem Cells
Source: Polymers (Basel). 2023 Apr 3;15(7):1777. doi: 10.3390/polym15071777 (PMC10098799; doi:10.3390/polym15071777)
Supplement: Supplementary file 1 [file polymers-15-01777-s001.zip › polymers-2263333-supplementary.pdf]

Supporting Information

One-pot synthesis of double-network PEG/collagen hydrogel for enhanced adipogenic differentiation and retrieval of adipose-derived stem cells

Hwajung Lee [a, †], Hye Jin Hong [a, †], Sujeong Ahn [a], Dohyun Kim [a], Shin Hyuk Kang [b], Kanghee Cho [a, *] and Won-Gun Koh [a, *]

[a] Department of Chemical and Biomolecular Engineering, Yonsei University, Seoul 03722, Republic of Korea
[b] Departments of Plastic and Reconstructive Surgery, Chung-Ang University Hospital, Chung-Ang University College of Medicine, Seoul 06973, Republic of Korea
† These authors contributed equally to this work
*Correspondence:
Won-Gun Koh, Ph.D. Department of Chemical and Biomolecular Engineering, Yonsei University, Seoul 03722, Republic of Korea; E-mail: wongun@yonsei.ac.kr; Contact: +82 2 2123 5755
Kanghee Cho, Ph.D. Department of Chemical and Biomolecular Engineering, Yonsei University, Seoul 03722, Republic of Korea; E-mail: jkh7t@hanmail.net; Contact: +82 2 2123 7795

S1. Chemical chracterization

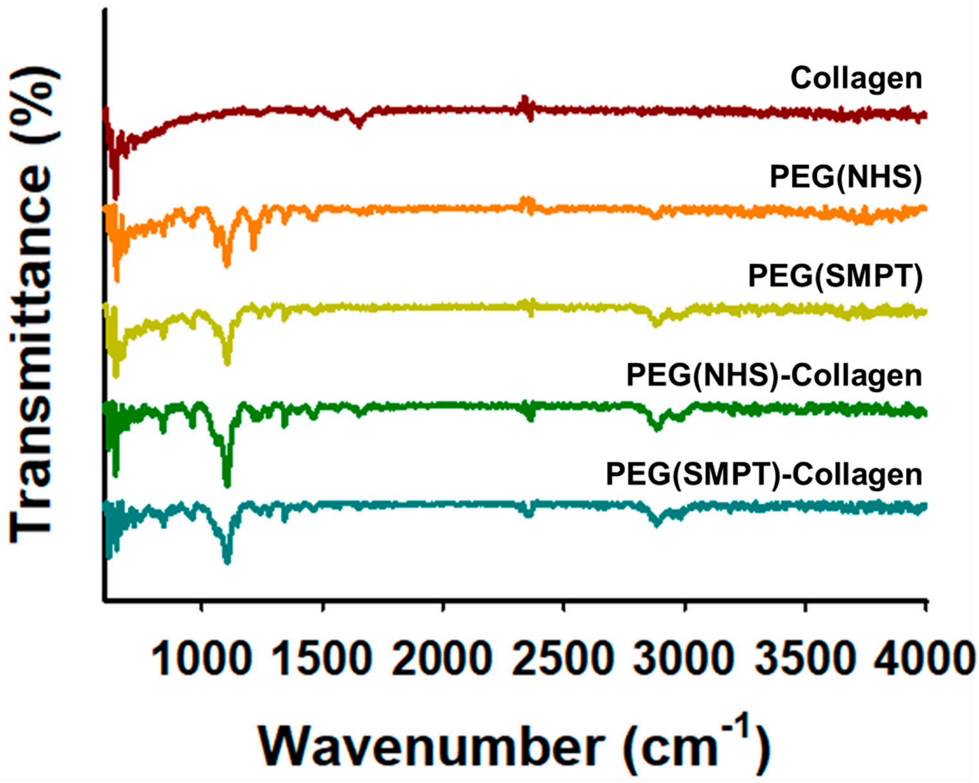

Figure S1. FTIR characterization of each hydrogel.

In the collagen hydrogel spectrum, two peaks originating from the amide I and amide II bands are clearly distinguishable. The amide I band at ~1650 cm^{-1} is caused by C=O stretching of the peptide carbonyl group, and the amide II band at ~1560 cm^{-1} is caused by NH_2 deformation in primary amides mixed with N-H bending and C-N stretching in secondary amides [1]. These bands become diminished as PEG is introduced and are absent in hydrogels without collagen.

In the spectrum of PEG(NHS), specific NHS bands at ~1210 cm^{-1} and ~1070 cm^{-1} were detected, indicating that unreacted NHS groups are slightly present [2]. This phenomenon may be due to the massive amount of NHS functionalities in the 4-arm-PEG-NHS molecules composing the PEG(NHS) hydrogel. When constructing other hydrogels, NHS groups in chemicals like SMPT, TCO, and MTz become totally reacted and consumed during attachment to amine-functionalized polymers prior to hydrogel formation.

On the other hand, PEG(SMPT) had no distinct peaks other than those originate from

PEG [3-5]. The strong peaks at 1100 cm^{-1}, ~1340 cm^{-1}, ~1470 cm^{-1}, and ~2880 cm^{-1} are attributed to PEG, representing C-H and C-O-C bands abundant in the PEG region. Minor bands under 1000 cm^{-1} are also observed from C-H vibration of PEG. The difference between the PEG(NHS)-Collagen and PEG(SMPT)-Collagen hydrogels is obscured by native PEG bands. In short, it is possible to distinguish the Collagen hydrogel, PEG(NHS) hydrogel, PEG(SMPT) hydrogel, and hybrid hydrogels (PEG(NHS)-Collagen and PEG(SMPT)-Collagen) from each other. However, the two hybrid hydrogels have analogous IR spectra. Distinguishing the two hybrid hydrogels from each other could be simple since the PEG(NHS)-Collagen hydrogel is not degraded by GSH, while the PEG(SMPT)-Collagen hydrogel is completely degraded (Figure 5b).

1. de Campos Vidal, B.;M. L. S. Mello, Collagen type I amide I band infrared spectroscopy, Micron **2011**, 42, 283-289.
2. Suys, O.;A. Derenne;E. Goormaghtigh, ATR-FTIR Biosensors for Antibody Detection and Analysis, Int. J. Mol. Sci. **2022**, 23, 11895.
3. Biswas, N.;A. J. Waring;F. J. Walther;R. A. Dluhy, Structure and conformation of the disulfide bond in dimeric lung surfactant peptides SP-B1–25 and SP-B8–25, Biochim. Biophys. Acta - Biomembr. **2007**, 1768, 1070-1082.
4. Taghavikish, M.;S. Subianto;N. K. Dutta;N. Roy Choudhury, Novel thiol-ene hybrid coating for metal protection, Coatings **2016**, 6, 17.
5. Sihota, P.;R. N. Yadav;V. Dhiman;S. K. Bhadada;V. Mehandia;N. Kumar, Investigation of diabetic patient’s fingernail quality to monitor type 2 diabetes induced tissue damage, Sci. Rep. **2019**, 9, 3193.
